# Thermostability of tetanus toxoid vaccine encapsulated in metal-organic frameworks

**DOI:** 10.1007/s13346-025-01838-4

**Published:** 2025-03-28

**Authors:** Rohan Murty, Krista S. Walton, Mark R. Prausnitz

**Affiliations:** School of Chemical and Biomolecular Engineering, Georgia Institute of Technology, Atlanta, GA USA

**Keywords:** Tetanus toxoid vaccine, Thermostability, Metal-organic frameworks, Zeolitic imidazolate framework, Biomimetic mineralization

## Abstract

Most vaccines require refrigerated transport and storage, which is costly, challenging in low-resource settings, and results in the loss of up to 50% of vaccines globally due to “cold-chain” failures. Here, tetanus toxoid vaccine (TT) was thermostabilized by encapsulation within a metal-organic framework (MOF), zeolitic imidazolate framework-8 (TT@ZIF-8). Its physicochemical properties were characterized by X-ray diffraction, Fourier transform infrared spectroscopy, scanning electron microscopy, and confocal microscopy. Unencapsulated TT fell below the 80% activity threshold within 4 days at 40˚C and 60˚C according to immunoassay analysis. Aqueous suspensions of TT@ZIF-8 also declined below 80% activity within a week at both temperatures, likely due to MOF degradation in water. Dried TT@ZIF-8 performed better, retaining 80% stability for 33 days at 40˚C and 22 days at 60˚C. When TT@ZIF-8 was suspended in a non-aqueous mixture of propylene glycol and ethanol, it remained 80% stable for approximately 4 months at 40˚C and 2.5 months at 60˚C. Arrhenius modeling predicted this formulation may qualify for “controlled temperature chain” designation, allowing partial vaccine removal from the cold chain. These studies suggest that MOF encapsulation of vaccines like TT can enable dramatic improvements in vaccine stability during storage without refrigeration.

**Graphical abstract:**

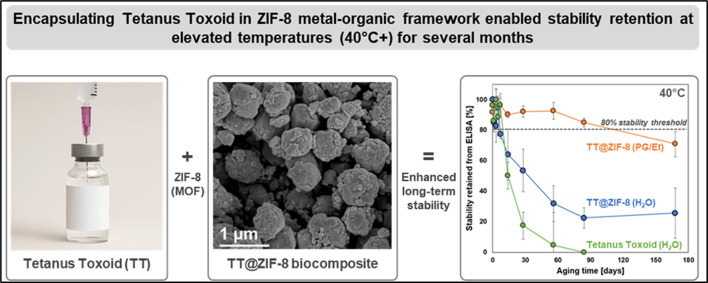

## Introduction

Immunization against infectious diseases is known to markedly improve financial, social, and health outcomes, especially in developing countries [1, 2]. While global vaccination rates for diseases like polio, measles and diphtheria-tetanus-pertussis (DTP) have reached 80–90% in the last three decades, there were more than 14 million children who received no vaccinations at all in 2022, and many millions more were only partially vaccinated [3, 4]. Most of these children live in regions of Africa and Asia that have high temperature and humidity, and unreliable electricity sources [5]. Because many vaccines are temperature-sensitive, they must often be stored at 4–8 °C, and short lapses in this refrigeration can cause irreversible vaccine damage [6]. Millions of doses are lost to failures in this “cold chain” annually, with the World Health Organization estimating that up to 50% of all vaccines are wasted globally [7, 8].

The dominant mechanism to stabilize vaccines and other biologics is by limiting molecular movement and interaction [9]. This can reduce conformational mobility of biomolecules in vaccines, thereby inhibiting their unfolding and denaturation. It can also slow chemical reactions (e.g., oxidation, hydrolysis) that can damage vaccines. This is why vaccines are stored under refrigeration, which slows molecular motion and chemical reactions. Freezing can immobilize molecules even more, but the freeze-thaw process can damage vaccines, and frozen storage is even more complex and costly in low-resource settings [9].

Lyophilization (i.e., freeze-drying) is also commonly used to stabilize temperature-sensitive vaccines, which locks biomolecules in a rigid matrix with very low water content [10, 11]. While this method has been studied extensively, there are several drawbacks including high cost, a necessary reconstitution step to re-dissolve the vaccine in water, and loss of activity during lyophilization and upon reconstitution [12–14]. The addition of excipients, either to liquid or dried formulations, is another common method for improving vaccine stability by stabilizing biomolecule structure and limiting intermolecular interactions, but these components must be carefully screened and tailored for each specific application, and often do not fully stabilize the vaccine [15, 16].

Here, we study a new approach to vaccine stabilization by encapsulating vaccine antigen in metal-organic frameworks (MOFs), a class of highly tunable porous materials composed of metal nodes and ligand linkers that can form a rigid matrix [17, 18]. Through a self-assembly process called biomimetic mineralization, a variety of proteins, viruses, and cells have been encapsulated within the rigid framework, imparting significant chemical and thermal stability to the entrapped molecules [19–21].

Because many MOFs lack water stability, require harsh synthesis conditions, and/or are composed of toxic metals and/or ligands, there are several barriers to safe applications of MOFs for medical applications [22–24]. However, the MOF species zeolitic imidazolate framework-8 (ZIF-8) is an attractive candidate for MOF encapsulation of biomolecules [25, 26]. This is because it can be synthesized under aqueous, room-temperature conditions, possesses stability in water on the order of days, and is composed of a zinc (II) metal center and 2-methyl imidazole ligand, which are expected to be biocompatible at relatively low concentrations [27–31]. ZIF-8 was selected as a model MOF over other possible host species due to its well-characterized nature and relatively high biosafety; nonetheless, other MOF species are also of interest for future study [32–34].

Prior studies have investigated the biomimetic mineralization of ZIF-8 around various biomacromolecules to improve thermostability of model proteins, viruses, and antigens such as bovine serum albumin (BSA), tobacco mosaic virus (TMV), and ovalbumin [35–39]. Herbert et al. demonstrated the encapsulation and protection of proteoliposomes (including in the liquid state) at elevated temperatures (e.g., 55˚C and 80˚C), but only examined their stability after exposure for up to 15 min [39]. Luzuriaga et al. showed TMV-ZIF biocomposite stability was retained after exposure to 100˚C for less than 1 h [30]. Teng et al. showed VLP-ZIF biocomposite stability was retained at 60˚C for 3 h. Other groups have shown TT encapsulated in a MOF [40], but did not examine thermal stability. Thus, the MOF literature has generally assessed thermostability of biocomposites using model compounds (i.e., not vaccines or therapeutics of direct clinical relevance) over short periods of time (e.g., hours). There is a need to study the stability MOF-encapsulated vaccines and therapeutics over longer periods of time (e.g., months) at elevated temperatures.

In this study, we assessed stability of tetanus toxoid (TT), one of the most-widely used vaccines in the world [41], encapsulated in ZIF-8 (i.e., TT@ZIF-8). TT vaccine specification is generally for a minimum dose, and product specifications allow significant variation in vaccine potency (e.g., 50–200%) [42]; here, we used commercial TT vaccine antigen from the Serum Institute of India (SII). Biocomposite stability was studied in both liquid and dried forms over timescales up to 6 months and at temperatures up to 120˚C without the use of additional excipients. TT was selected for our study due to its ubiquity, translational relevance, well-characterized nature, and lack of stability after weeks at temperatures greater than 40˚C [9]. Spectroscopic, crystallographic, and microscopic techniques were used to first confirm the encapsulation of TT within the ZIF-8 framework and then to characterize the resulting composite material. Accelerated stability studies were conducted at 40 °C and 60 °C using dried TT@ZIF-8 powder as well as two different liquid suspensions of TT@ZIF-8. We also examined biocomposite thermostability over several freeze/thaw cycles and at an extreme temperature of 120˚C. The overall goal of this work was to characterize the long-term thermostability (and thus, shelf life) of a clinically relevant vaccine-MOF composite in a variety of harsh storage conditions, ultimately suggesting vaccine-ZIF formulations as a viable approach for breaking the cold chain.

## Materials and methods

### Materials

Formalin-inactivated TT vaccine antigen was kindly provided by SII (Pune, India). The composition of the stock solution was reported previously [43]. fTT stock was prepared by tagging TT with Alexa Fluor 488 according to the protocol provided in the Alexa Fluor 488 protein labeling kit (Thermo Fisher) [44].

100x anti-tetanus toxoid horse radish peroxidase (HRP) conjugate antibody (Alpha Diagnostic International, San Antonio, TX, USA), KPL SureBlue 3,3’,5,5’-tetramethylbenzidine (TMB) Microwell Substrate (Seracare, Milford, MA, USA), KPL TMB BlueSTOP (Seracare), zinc (II) acetate dihydrate (≥ 98%, Sigma-Aldrich, St. Louis, MO, USA), zinc (II) nitrate hexahydrate (98%, Sigma-Aldrich), 2-methyl imidazole (≥ 98%, TCI, Tokyo, Japan), triethylamine (98%, Sigma-Aldrich), propylene glycol (> 99%, Sigma-Aldrich), ethanol (90%, Sigma-Aldrich), 10x phosphate-buffered saline (> 99%, Growcells, Irvine, CA, USA), EDTA (99%, Sigma-Aldrich), Immersol (Carl Zeiss, Oberkochen, Germany), and Drierite (Lab grade, Alfa Aesar, Haverhill, MA, USA) were used as received from the manufacturer.

Deionized (DI) water was supplied by an in-house system (Thermo Fisher, Waltham, MA, USA). Tycho NT.6 Capillaries (NanoTemper Technologies, München, Germany) were used for differential scanning fluorimetry experiments.

### Synthesis of MOF and biocomposite

#### Room-temperature synthesis of ZIF-8

The synthesis procedure was followed from the work of Gross et al. [29]. ZIF-8 structures with a 1:16:16 ratio of metal: ligand: triethylamine were prepared by first dissolving 0.733 g zinc (II) nitrate (2.46 mmol) in 50 mL DI water. Then, a solution of 3.244 g of 2-methyl imidazole (39.5 mmol) and 4.00 g TEA (39.52 mmol) in 50 mL DI water was stirred until dissolved. The zinc salt solution was added to the 2-methyl imidazole/TEA solution, and the resulting mixture was stirred for 10 min to form ZIF-8 particles. This mixture was then separated via centrifugation (3,000 x g 10 min), decanted, and suspended in DI water for 12 h. This centrifugation process was repeated for a second water wash. After 12 h, the ZIF suspension was centrifuged again, and the solid was collected. Solid ZIF crystals were finally dried under vacuum for 2 h at 150℃.

#### Room-temperature synthesis of TT@ZIF-8

The synthesis procedure was adapted from the work of Liang et al. [20]. TT@ZIF-8 structures were synthesized by preparing 20 mL of two solutions, one containing 160 mmol ligand solution of 2-methyl imidazole and the other containing 40 mmol of zinc (II) acetate. One milliliter of the TT vaccine stock (3,480 Lf or roughly 10 mg) was added to the 2-methyl imidazole solution a few minutes before addition of the zinc solution. Upon addition of the metal, solid particles appeared instantly and were agitated for 1 min; the combined solution was then left for 16 h. The same procedure was followed to synthesize fTT@ZIF-8. The resulting biocomposite crystals were separated from solution by centrifugation (3,000 x g, 10 min) and then washed, sonicated, and centrifuged twice each with water and ethanol. The resulting pellet was dried at ambient conditions (20–25˚C, 30–60% relative humidity) for 5–7 days before weighing; the average yield was ~ 90 mg of TT@ZIF-8.

### Assessment of TT@ZIF-8 biocomposite structure

#### Confirming crystallinity by powder X-ray diffraction

MOF crystal structure was confirmed with pXRD. Measurements were performed on the XPert Pro Alpha-1 diffractometer with X’Celerator detector using Cu Kα_1_ radiation (λ = 1.540598 Å) (Malvern Panalytical, Malvern, United Kingdom). A 2θ range of 5° to 70° was measured using a step size of 0.0167° 2θ and a time per step of 10.16 s. Scherrer analysis of crystal size was conducted in the HighScore Plus program (Malvern Panalytical).

#### Assessing TT incorporation into ZIF-8 framework by Fourier-transform infrared spectroscopy

FTIR measurements were done on a Nicolet iS10 FTIR spectrometer with the Smart iTX diamond ATR accessory (Thermo Fisher). Pure ZIF-8 MOFs, biocomposites, and lyophilized TT were all analyzed as dry powders. We performed 64 scans with a resolution of 2 cm^-1^.

#### Visualizing particle surface by scanning electron microscopy

Since the zinc-based ZIF-8 is non-conductive, all samples were prepared by gold sputtering (Hummer 6 sputterer, Ladd Research, Williston, VT) for 3–4 min before visualization by SEM (SU8230, Hitachi, Tokyo, Japan). Samples were observed at magnifications ranging from 5,000x to 25,000x at voltages ranging from 5.0 kV to 10.0 kV, and with a working distance of 9,061 μm.

#### Visualizing TT distribution within ZIF-8 structure by confocal microscopy

fTT@ZIF-8 powder was prepared for confocal imaging by suspension in Immersol. Confocal images were taken on a Zeiss LSM 700 (Carl Zeiss) with an objective lens of 63x. For z-stacked images, an interval of 0.200 μm was used for appropriate resolution imaging of ~ 1 μm fTT@ZIF-8 particles. Image processing and analysis were conducted in ZEN lite (Carl Zeiss).

### Measuring melting point of TT

The melting temperature of the stock TT was measured by differential scanning fluorimetry on a Prometheus NT.48 (NanoTemper Technologies). The stock vaccine solution was diluted to a concentration of 10 µM TT before measurement, and a ramp rate of 1 °C/min was used over a temperature range of 20 °C to 90 °C. Samples were measured in triplicate.

### Preparation of TT and TT@ZIF-8 for stability studies

#### Dried and liquid TT samples

For liquid-phase stability experiments, TT stock solution was diluted to 100 µg/mL in DI water and aliquoted in glass vials. For solid-phase stability experiments, TT stock solution was first diluted to 1 mg/mL in DI water before placing in a -80 °C freezer. The frozen vaccine was then lyophilized overnight (Labconco, Kansas City, MO) under a vacuum of 0.700 mbar. For unformulated TT vaccines solvated in a 4:1 mixture of propylene glycol and ethanol (PG/Et), 20 µL of a 100 µg/mL TT stock solution was added to 980 µL of the PG/Et mixture to create a 2 µg/mL dilution of TT in solvent.

#### Dried and suspended TT@ZIF-8 samples

Dried TT@ZIF-8 was packaged in glass vials in ambient conditions before sealing the vials in aluminum pouches containing color-indicating desiccant (Drierite). Upon removal of vials from pouches, the desiccant largely retained its blue color, suggesting a lack of significant moisture. Suspensions of TT@ZIF-8 were prepared at a concentration of 1 mg/mL in two solvents: DI water and a 4:1 mixture of PG: Et. After packaging, vials containing liquid samples were placed in a sand bath during thermal exposure to mitigate temperature fluctuations.

#### Freeze/thaw samples

A 1 mg/mL suspension of TT@ZIF-8 in DI water was stored in a glass vial in a -80 °C freezer for 20 min before removal and placement in a room-temperature water bath for 15 min. This process was cycled up to 5 times.

### Measurement of encapsulated/released TT concentration

#### Dissolution of ZIF-8 structure

At various time points during stability studies, ZIF-8 structures were removed from TT@ZIF-8 biocomposites using a procedure adapted from Li et al. [45]. A volume of 62 µL of a 0.1 M solution of EDTA (pH 6.9–7.0) was added to 1 mg TT@ZIF-8 powder, corresponding to an approximate molar ratio of 3:2 EDTA: TT@ZIF-8. EDTA was added directly to liquid suspensions, and solid samples were suspended in water at a concentration of 1 mg/mL before immediate addition of EDTA. Samples were left to incubate for 24 h at room temperature before measuring retained TT activity. After dissolution of the MOF scaffold with EDTA, samples were diluted and measured with ELISA without removal of EDTA or Zn-EDTA residues, as specific precautions were taken to ensure the EDTA concentration was below the threshold of interference with Abcam’s ELISA protocol (1 mM EDTA) [46]. EDTA concentration in samples was ~ 6 mM before dilution for ELISA by at least 50-fold, ensuring the final concentration was significantly less than 1 mM.

#### Measuring TT activity

Activity of TT was measured with direct, competitive ELISA, using TT epitope binding as a surrogate measure that may correlate with TT vaccine potency [47]. A monoclonal anti-TT antibody was used in this study. Although the specific epitope binding site was not available from the antibody manufacturer (ADI), previous studies from our group have shown a strong correlation between the amount of TT detected by the same competitive ELISA measurement and the antigen-specific antibody titers and neutralizing activity in vivo [43, 48].

After coating plates with TT antigen, liberated samples and standards were diluted to the appropriate concentration range (30–2000 ng/mL). Each antigen-containing sample was then mixed in an equal volumetric ratio with anti-TT HRP antibody and incubated at 37 °C for 1 h. After adding the sample-antibody mixtures, the plate was incubated overnight at 4 °C. After addition of TMB substrate and stop solution, concentrations were measured with an iMark Microplate Absorbance Reader (Bio-Rad, Hercules, CA, USA) at a wavelength of 620 nm.

#### Calculating encapsulation/release efficiency of TT within ZIF-8

Using ELISA, the concentration of unencapsulated TT was measured by collecting the supernatant from each wash cycle on the freshly prepared particles. These values were then examined alongside the amount of TT released from the ZIF after liberation to close the following mass balance:

$$\begin{aligned}T{T}_{total}&=T{T}_{\left[{H}_{2}O\:washes\right]}+T{T}_{\left[EtOH\:washes\right]}\\&+T{T}_{released}+T{T}_{unreleased}\end{aligned}$$

where TT_total_ is the amount of TT used to the synthesis of TT@ZIF-8; TT_[H2O washes]_ and TT_[EtOH washes]_ are the amounts of TT recovered in the supernatant after the water and ethanol washes during the synthesis, respectively; T_treleased_ is the amount of TT measured after dissolution of ZIF-8 from TT@ZIF-8; and TT_unreleased_ is the amount of TT that was not released after dissolution of ZIF-8 from TT@ZIF-8 (or not detected when measurement of the other TT groups was performed, possibly due to loss of TT activity upon release from the framework as measured by ELISA).

### Modeling TT@ZIF-8 stability with the arrhenius relationship

Exponential regressions were first performed on the data presented in Fig. 4, accounting for errors in stability measurements (see below). These fits allowed forecasting of the days to failure, defined as losing 20% of the initial TT activity measured by ELISA. The number of days to failure (a proxy for the reaction rate) at 40˚C and 60˚C were used to generate an Arrhenius equation (shown below), which was then used to predict stabilities at lower temperatures:

$$\:k=A{e}^{\left(-\frac{{E}_{a}}{RT}\right)}$$

In this equation, k is the rate of stability loss, A is the pre-exponential factor, E_a_ is the activation energy of the reaction, R is the ideal gas constant, and T is storage temperature.

Error was propagated through two functions—the exponential regression and the Arrhenius fit. Variance in calculations for days to failure was generated by creating several fits of the data (based on the errors in the stability measurements) and analyzing the statistical distribution. The corresponding days to failure from these various fits were then inputted into 1,000,000 possible Arrhenius equations (which varied based on the initial input of days to failure). We then reported the means and standard deviations of the outputs of the Arrhenius model. In this way, the error of the stability measurements was propagated throughout both calculations. Three key assumptions underlie this model: (i) that no studied condition (at 40˚C or 60˚C) would retain 80% stability for 10 years, (ii) distribution of errors around stability measurements are Gaussian in log space, and (iii), stability will never increase between subsequent time points (i.e., maximum stability occurs at *t = 0*).

### Statistical analysis

Statistical analysis and generation of p-values for the accelerated stability study (Fig. 4) were completed with an online calculator (www.statskingdom.com) for one-way ANOVA, with a significance threshold of *p* < 0.05 [49]. A detailed description of the statistical methods used in the Arrhenius modeling can be found in the immediately preceding section.

## Results

### Encapsulation of tetanus toxoid in ZIF-8

**Fig. 1:**
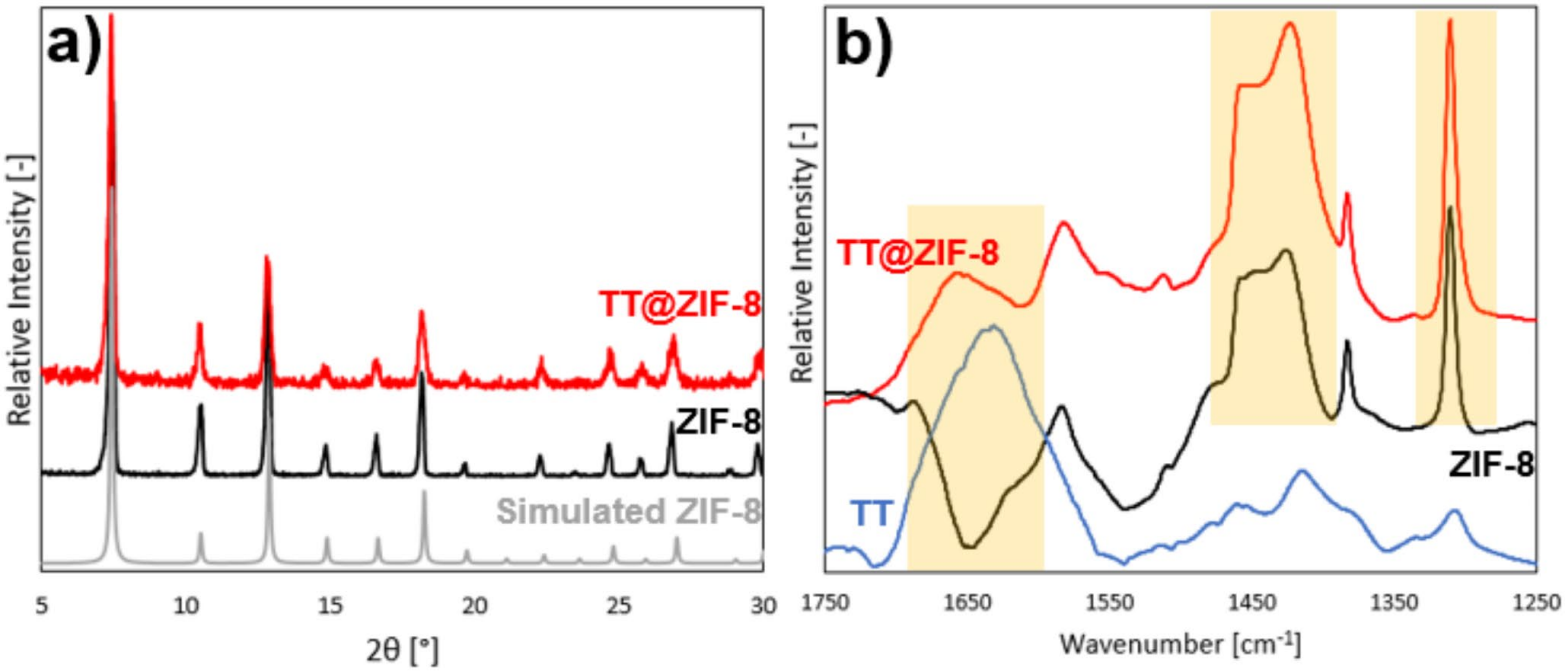
Crystallographic and spectroscopic characterization of TT@ZIF-8 biocomposite, pure ZIF, and lyophilized TT. (**a**) Representative powder X-ray diffraction spectra of TT@ZIF-8 biocomposite (red), pure ZIF-8 structure (black), and simulated ZIF-8 structure (gray, from [50]). (**b**) Representative FTIR spectra of TT@ZIF-8 biocomposite (red), pure ZIF-8 structure (black), and lyophilized TT vaccine (blue)

We formed TT@ZIF-8 biocomposites by mixing TT with zinc and 2-methyl imidazole in aqueous solution at room temperature for 16 h. The resulting structures were characterized by powder X-ray diffraction (pXRD) and Fourier transform infrared spectroscopy (FTIR) to confirm the material structure and presence of TT within the ZIF-8 scaffold (Fig. 1).

pXRD diffractograms show that the synthesized ZIF-8 structure (black spectrum in Fig. 1a) is in excellent agreement with previously simulated crystallographic data for the ZIF-8 sodalite topology (gray spectrum in Fig. 1a) [50]. The presence of sharp peaks at 2θ values of 7.50°, 10.5°, and 13.0° suggest that the pure ZIF MOF was successfully synthesized. The biocomposite diffractogram (red spectrum in Fig. 1a) matched the peak locations and relative intensities shown for ZIF-8, suggesting that the ZIF structure was formed in the presence of TT. The biocomposite diffractogram also exhibited a larger signal-to-noise ratio than that for pure ZIF-8, likely owed to disruption of the ZIF’s long-range order by the large (150 kDa) TT molecules embedded within the framework. We conclude that these pXRD results demonstrate that encapsulating TT within ZIF-8 did not significantly alter the crystalline structure of the framework.

After confirming the crystalline structure of TT@ZIF-8, the chemical effects of TT incorporation into the framework were studied using attenuated total-reflection (ATR) FTIR (Fig. 1b). The lyophilized TT vaccine notably contains a broad Amide I peak from 1700 to 1550 cm^-1^ (blue spectrum in Fig. 1b), owed to interactions between acyl groups (R—C = O) and nitrogen atoms [51]. The lyophilized TT spectrogram also contains a much smaller peak in the Amide III region (1350–1250 cm^-1^), which is in agreement with previous work on lyophilized TT vaccine [13, 52].

The prominent Amide I peak observed in TT is notably missing from the pure ZIF spectrogram (black spectrum in Fig. 1b) since the MOF is composed only of zinc metal centers and 2-methyl imidazole ligands. While ZIF-8 does show a sharp peak at 1310 cm^-1^ that coincides with the TT Amide II peak, this is not owed to any proteinaceous behavior, but is rather due to the C—N stretching mode from the aromatic amine structure of the 2-methyl imidazole ligand [51]. The primary characteristic feature of ZIF-8 is the plateau ranging from 1460 to 1390 cm^-1^ caused by C—H bending from the ligand [20], which is absent from the TT spectrum.

As expected, the TT@ZIF-8 spectrogram contains both the Amide I peak from the TT molecules as well as the characteristic ZIF-8 plateau and strong C—N stretching peak of ZIF-8 (red spectrum in Fig. 1b). The Amide I peak shifts upwards slightly in the TT@ZIF-8 biocomposite, possibly due to interactions between TT and ZIF-8; this is consistent with the hypothesis that TT is encapsulated within the ZIF-8 structure, but does not rule out the possibility that it is surface-bound. Other peaks and features of the ZIF-8 and TT@ZIF-8 biocomposite spectra agree with previous similar studies on various encapsulated molecules such as BSA and glucose oxidase [20, 53].

### Imaging TT@ZIF-8 biocomposites

**Fig. 2:**
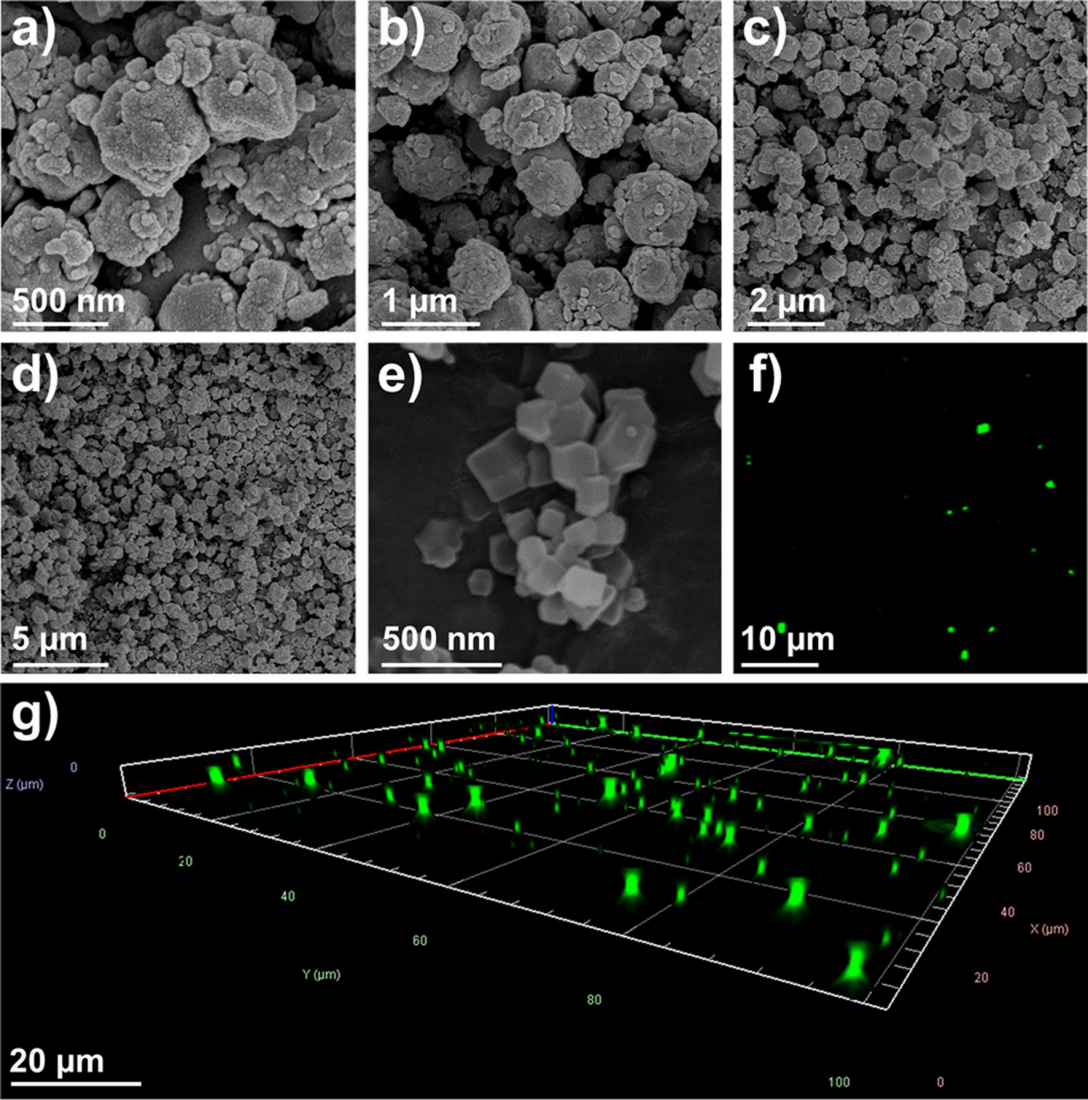
Representative microscopy images of TT@ZIF-8 biocomposite: (**a**–**d**) Series of scanning electron micrographs of as-synthesized TT@ZIF-8 particles at decreasing magnification levels, (**e**) Scanning electron micrograph depicting ZIF-8 particles synthesized in aqueous, room temperature conditions, (**f**) Confocal fluorescence micrograph of fluorescently tagged fTT@ZIF-8, and (**g**) Z-stack image from confocal microscopy of fTT@ZIF-8 particles (where the full Z-axis represents a height of 4 μm)

Microscopy studies were conducted on the TT@ZIF-8 biocomposites in order to determine average particle size and assess distribution of TT within the ZIF structure (Fig. 2).

Scanning electron microscopy (SEM) imaging depicted a wide range of particle sizes with edge lengths ranging from 50 nm to 700 nm (Fig. 2a and d**)**. Notably, the TT@ZIF-8 particles did not appear to assume the sharp, rhombic dodecahedra geometry often associated with ZIF-8 biocomposites [20, 54, 55]. In contrast, when pure ZIF-8 was synthesized under similar (i.e., aqueous, room-temperature) conditions as TT@ZIF-8, the particle shapes appeared significantly more regular (Fig. 2e). Smaller crystals appear on the surface of the TT@ZIF-8 particles more frequently than they did for pure ZIF-8, which could have contributed to the wider size distribution of TT@ZIF-8 particles. This may be explained by the mechanism of ZIF biomimetic mineralization, which is driven by certain acidic amino acid residues on the protein surface aiding nucleation of framework growth around the encapsulated molecule [56]. Thus, the presence of TT during ZIF-8 synthesis could explain the variation in particle growth/geometry. Previous reports on ZIF biocomposites have shown wide variation in particle geometry dependent on the encapsulated molecule [20, 53].

Visualization of the fluorescently tagged biocomposite (fTT@ZIF-8) with confocal microscopy further indicated that TT was distributed throughout the ZIF particles (i.e., appearing with uniform green color throughout the TT@ZIF-8 structure in Fig. 2f and g) instead of localized on the surface (i.e., not appearing as bright rings around a dark core).

Each green dot in Fig. 2f represents a particle aggregate. The size of a particle aggregate appears by visual inspection to be generally consistent with the higher-resolution images generated from SEM experiments (Fig. 2a and d). Z-stack images of the fluorescent particles (Fig. 2g**)** further underscored that TT molecules were present throughout the biocomposite structure and not just on the surface. This finding is consistent with a previous study on fluorescently-tagged proteins encapsulated within ZIF-8 [55].

### Quantifying TT@ZIF-8 stability in harsh storage conditions

#### Release of TT from ZIF structure

We hypothesized that encapsulation of TT in rigid ZIF-8 frameworks would stabilize TT in harsh storage conditions. Before characterizing stability during storage, we first assessed possible loss of TT during the process of encapsulation of TT in ZIF-8, followed by TT release from the biocomposite by ZIF dissolution using ethylenediaminetetraacetic acid (EDTA).

**Fig. 3:**
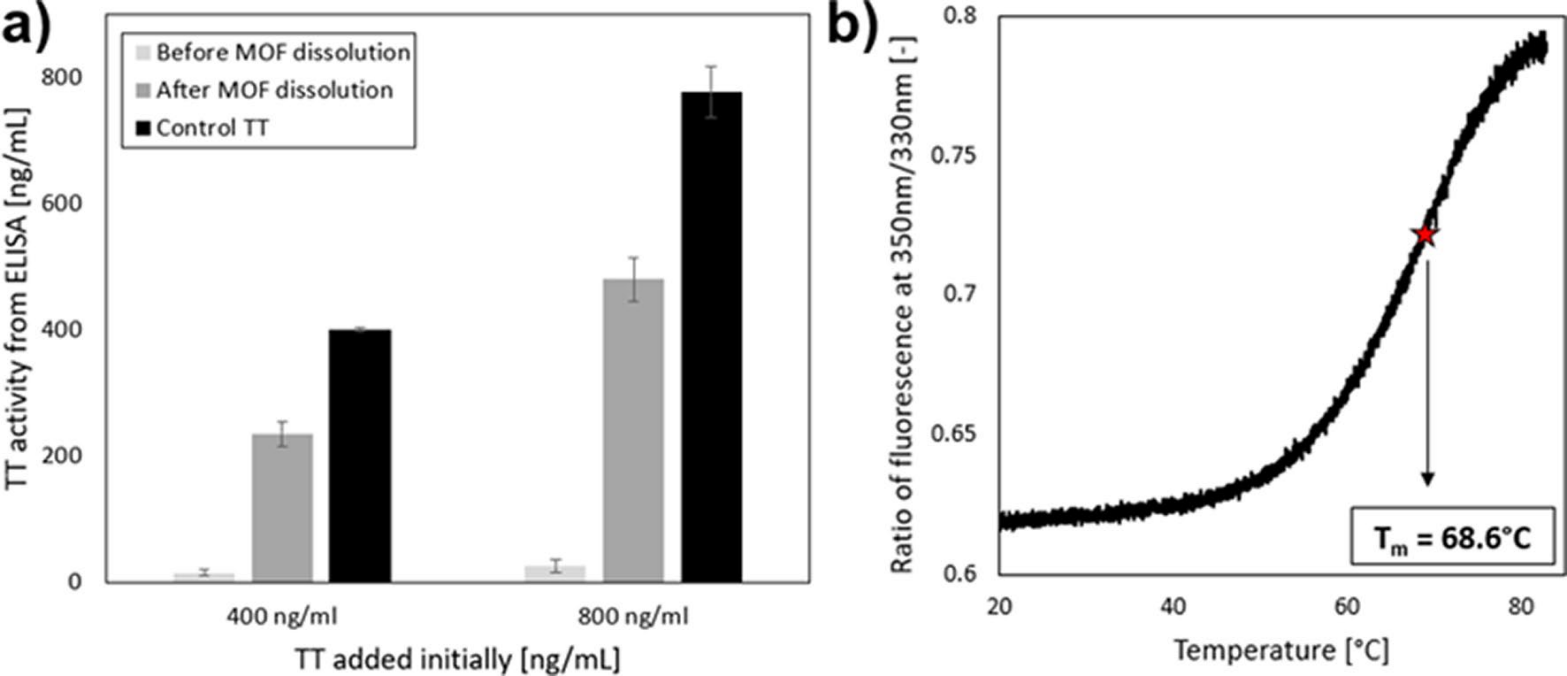
Characterization of tetanus toxoid (TT). (**a**) TT activity is compared between three types of samples: when TT is encapsulated within the TT@ZIF-8 biocomposite (i.e., before MOF dissolution), when TT has been liberated from the MOF structure (i.e., after dissolution), and before TT is incorporated into the MOF structure (i.e., free-standing TT in solution). Activity was measured by direct competitive ELISA. Data represent the average ± standard deviation (*n* = 3). (**b**) Differential scanning fluorimetry of 10 µM TT in water (data were measured with a ramp rate of 1 °C/min, *n* = 3)

When TT activity associated with intact TT@ZIF-8 was measured, a minimal signal was detected, because most TT was encapsulated in the interior of the ZIF-8 MOF and was therefore inaccessible for binding in the enzyme-linked immunosorbent assay (ELISA) used to assess epitope binding activity of the TT, which has been shown to be a good in vitro correlate with in vivo TT vaccine potency [47] (Fig. 3a). The small signal that was measured was likely due to residual TT on the particle surface not removed during the washing steps.

After particle dissolution to release the encapsulated TT, much greater activity was measured, but it was still less than the TT activity before encapsulation (comparison between dark gray and black bars in Fig. 3a). This could be explained by damage to TT during encapsulation and release, and/or incomplete release of TT from ZIF-8 particles. Loss of TT activity during biocomposite dissolution could be owed to the chelating agent EDTA, which is known to associate with and alter properties of proteins [57]. Future studies on the effects of EDTA on TT concentration are required for a full understanding of how all components in a potential TT-MOF vaccine formulation affect antigenicity. Control experiments to assess possible interference of ZIF-8 (or its constituents) or of ETDA with the ELISA measurement were not performed. However, any possible interference effect would be consistent across measurements, as the amount of ZIF-8 and EDTA were the same in all prepared samples. Nevertheless, TT recovery efficiency was approximately 60%, which is consistent with previous studies that measured release efficiencies of 60–70% [30].

The encapsulation efficiency of TT within ZIF-8 was found to be approximately 80%. This value was quantified by subtracting the amount of TT in surface wash supernatants and the surface-bound TT from the initial amount of TT added (as described in Materials and Methods). For example, when 5 mg of TT was introduced to the biomimetic mineralization reaction, measured TT concentrations in the washes by ELISA were: 66 ± 10 µg in the supernatant, 46 ± 0.6 µg in the first water wash, 50 ± 0.7 µg in the second water wash, 158 ± 19 µg in the first ethanol wash, and 358 ± 5 µg in the second ethanol wash. From Fig. 3a, 5–10% of the TT not removed in the surface wash (approximately 324 ± 153 µg) remained bound to the surface of the crystallites. In total, the non-encapsulated TT summed to 1,002 ± 188.3 µg (i.e., 1 mg for every 5 mg TT introduced or ~ 20%), indicating that 80% was successfully encapsulated within the MOF structure. 

### Stability of TT@ZIF-8 during extended storage at elevated temperatures

After performing these characterization studies, we next assessed the core hypothesis of the study by measuring the long-term stability of TT@ZIF-8 composites in liquid and dry states for up to 6 months at elevated temperatures of 40 °C and 60 °C. These temperatures were selected to enable accelerated testing of TT stability at temperatures at which ZIF-8 is known to be stable [58, 59] and below the melting point (T_m_ = 68.6 °C) of TT, as measured by differential scanning fluorimetry (Fig. 3b), in agreement with previous work [60]. This approach enabled extrapolation of stability data to lower temperatures *via* the Arrhenius relationship [61].

**Fig. 4:**
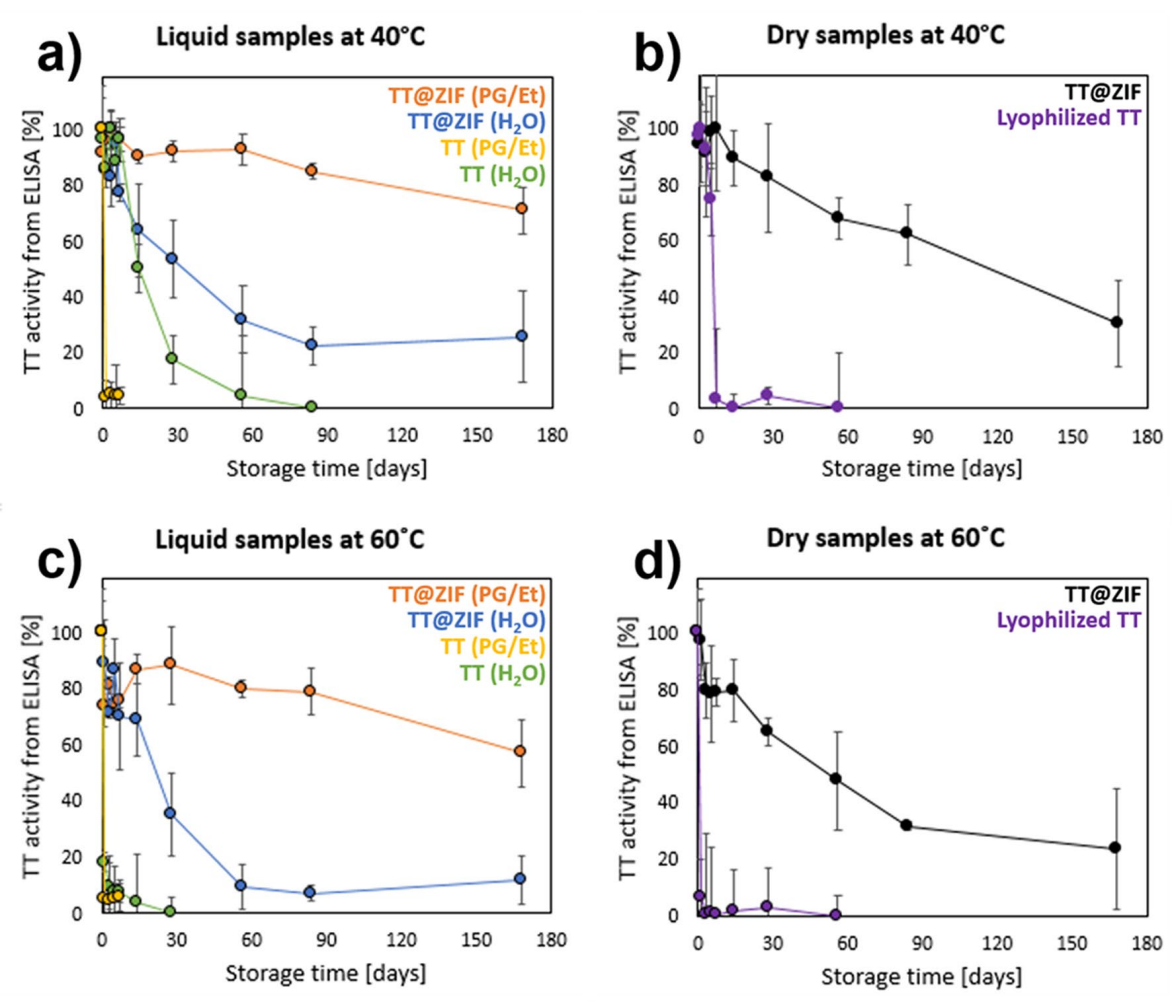
Stability of TT@ZIF-8 during long-term storage at elevated temperature. TT activity was determined during storage at (**a**, **b**) 40 °C and (**c**, **d**) 60 °C. TT@ZIF-8 was stored (**a**, **c**) in liquid state as a suspension in water or a mixture of polyethylene glycol and ethanol (PG/Et) or (**b**, **d**) as a dry powder. TT activity was measured by direct, competitive ELISA, and was normalized to the amount of recovered TT for each freshly made formulation. Freshly made TT@ZIF-8 suspended in PG/Et had a TT activity that was 43 ± 9% of freshly made TT@ZIF-8 samples suspended in water. All data represent the average ± standard deviation (*n* = 3)

When TT vaccine was stored in water, it lost essentially all activity after 8 weeks at 40 °C (Fig. 4a) and after just 2 weeks at 60 °C (Fig. 4c). These kinetics are consistent with a previous study that showed full activity loss in 6 weeks at 45 °C [62]; however, other studies report greater thermostability of the TT vaccine when formulated with adjuvants that can stabilize the vaccine [9, 63]. Indeed, with addition of an adjuvant, the DTP vaccine retained ~ 60% potency after exposure to 45 °C for 8 weeks, but lost all activity after a few hours of exposure to 60 °C [9].

**Fig. 5:**
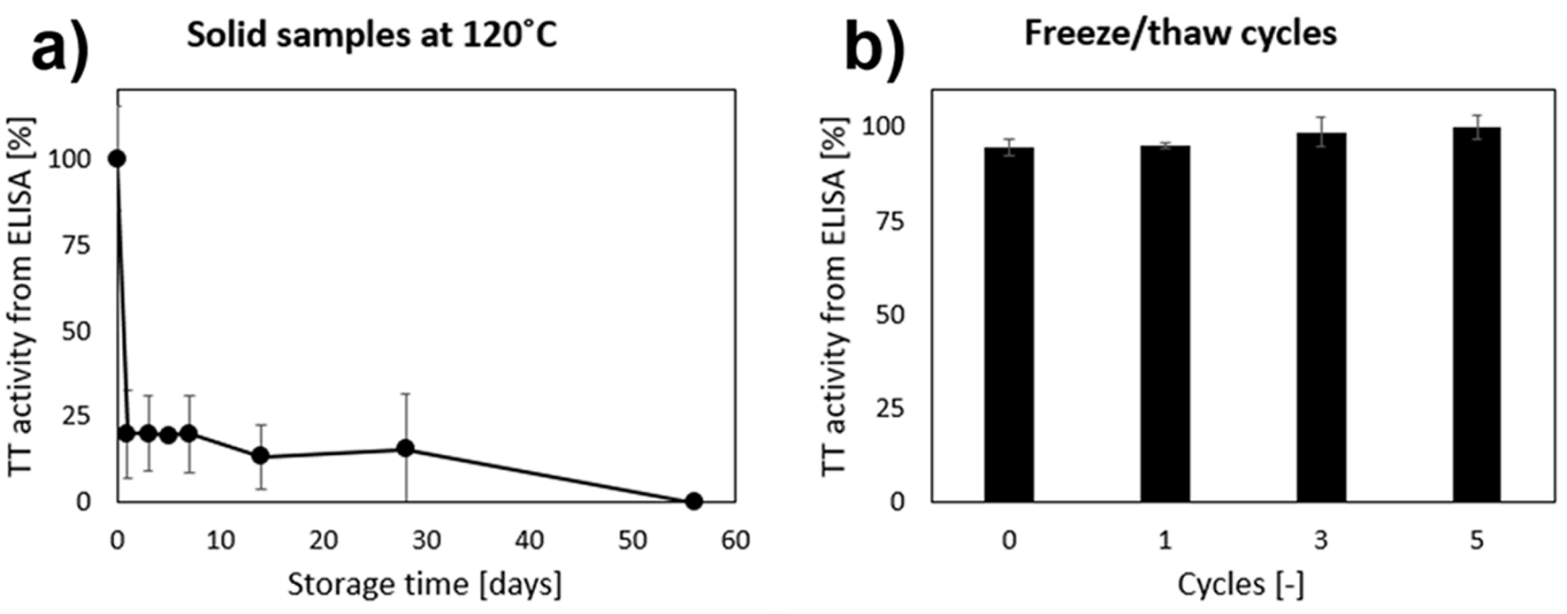
TT@ZIF-8 stability after exposure to extreme temperature and repeated freeze/thaw cycles. Storage of (**a**) dried TT@ZIF-8 powder at 120 °C and (**b**) aqueous suspension of TT@ZIF-8 during freeze/thaw cycles. Activity was measured by direct, competitive ELISA. Data represent the average ± standard deviation (*n* = 3)

TT@ZIF-8 in water also exhibited rapid activity loss at first, but then stabilized, retaining 26% ± 16% activity at 40 °C (Fig. 4a) and 12% ± 9% at 60 °C (Fig. 4c) after almost 6 months. This loss of stability may be explained by the instability of ZIF-8 in liquid water, which can be hydrolyzed [28], causing the release of encapsulated TT into the unstable aqueous environment. These findings suggest that TT@ZIF-8 suspension in water may not be a viable approach for long-term vaccine storage at elevated temperatures.

We therefore wanted to suspend TT@ZIF-8 in a non-aqueous liquid that would not dissolve or damage the biocomposite structure and would be biocompatible with tissues in the body. We selected a 4:1 mixture of propylene glycol and ethanol (PG/Et), which involves solvents that are safely used in pharmaceutical formulations [64], are not expected to degrade the ZIF framework [50], can be formulated with a density that matches the density of TT@ZIF-8 (i.e., 0.94 g/mL, to maintain a uniform suspension without settling or floating), and does not interfere with the ELISA measurements.

We found that PG/Et was a good solvent for stabilizing TT@ZIF-8. After 6 months at elevated temperatures, these suspensions retained 71% ± 8% activity at 40 °C (Fig. 4a) and 57% ± 12% activity at 60 °C (Fig. 4c), representing a marked improvement over TT@ZIF-8 storage in water (Fig. 4a; one-way ANOVA, *p* < 0.001 at both temperatures). This approach exploited the much higher relative stability of ZIF-8 in organic solvents as opposed to water [20, 28, 50].

In contrast, lyophilized TT dissolved in the PG/Et solution completely lost activity after just 1 day at both 40 °C and 60 °C (Fig. 4a and c) and was significantly less stable than TT in water at 40 °C (Fig. 4a; two-way ANOVA, *p* < 0.001), which is likely due to the lower stability of TT in organic solvents [65] and the presence of residual water in the PG/Et mixture [66]. This indicates that PG/Et was not a good solvent for stabilizing TT, but was a good solvent for stabilizing ZIF-8, in which TT was encapsulated. This difference is at least partially owed to the fact that TT encapsulated within ZIF-8 resides within conformally-sized cavities, preventing the unfolding (i.e., denaturation) of the encapsulated protein [67], whereas free-standing TT in the solvent can be damaged as it interacts with a mixture of organic solvents and water. 

Because vaccines and other biologics are more stable in a dried state than in liquid, we also determined TT stability as a dry powder. Lyophilized TT was remarkably unstable, losing essentially all activity within one day at 40 °C and 60 °C (Fig. 4b and d), which was similar to lyophilized TT dissolved in PG/Et (Fig. 4a and c; one-way ANOVA, *p* = 0.53 at 40 °C). This suggests that PG/Et may not be damaging to lyophilized TT, and the rapid stability loss of lyophilized TT in PG/Et was mostly due to the instability of lyophilized TT.

After one month of storage, lyophilized TT powder (Fig. 4b) performed significantly worse than TT dissolved in water at 40 °C (Fig. 4a; one-way ANOVA, *p* < 0.001)). This may be explained by moisture-induced aggregation in lyophilized TT caused by residual formaldehyde stored in linkages from the process of creating the TT toxoid from the tetanus toxin [68].

In contrast, dried TT@ZIF-8 powder retained 30% ± 15% activity at 40 °C (Fig. 4b) and 24% ± 21% at 60 °C (Fig. 4d). This biocomposite stabilization represents a marked improvement when compared to the lyophilized vaccine after 2 months of storage (Fig. 4b and d; one-way ANOVA, *p* < 0.001 at both temperatures). This may be because lyophilized TT molecules are able to aggregate, causing irreversible potency loss, whereas TT molecules encapsulated within the ZIF-8 structure are sequestered in conformally sized cavities, which blocks intermolecular interactions between TT molecules, making them much less likely to aggregate [20, 67, 68].

Dried TT@ZIF-8 powder (Fig. 4b and d), however, was not as stable as TT@ZIF-8 suspended in PG/Et after 6 months of aging (Fig. 4a and c; two-way ANOVA, *p* < 0.001 at both temperatures). This is likely explained by the higher stability of ZIF-8 in organic solvents as opposed to water [28, 50]. The dried TT@ZIF-8 formulation may have been in contact with residual moisture from the air, causing hydrolysis of the structure [69], whereas the PG/Et suspension of the biocomposite limited the host framework’s contact with water molecules.

Finally, we examined the stability of dried TT@ZIF-8 powder at an extreme temperature of 120 °C and liquid suspension of TT@ZIF-8 in water through 5 freeze/thaw cycles (Fig. 5). The dried biocomposite retained 15% ± 16% stability for 1 week at 120 °C (Fig. 5a) and full activity loss was observed within 8 weeks (Fig. 5a). By this time, the original white TT@ZIF-8 powder had turned a blackish brown color, indicating chemical changes and possible degradation of the framework. This is a notably lower apparent decomposition temperature than for pure ZIF-8, which shows significant carbonization beyond 200 °C [70]. When exposed to 120 °C, unencapsulated TT lost essentially all activity in less than one day (data not shown).

When TT@ZIF-8 suspended in water was subjected to several freeze/thaw cycles, there was no significant loss of TT activity after 5 cycles (Fig. 5b; one-way ANOVA, *p* < 0.01). This suggests that while the aqueous formulation was not well-suited for long-term storage at high temperatures, it retained stability during freezing and thawing.

### Modeling TT@ZIF-8 stability with the arrhenius relationship

Data from accelerated testing of vaccine stability at high temperature can be used to predict stability at lower temperatures using the Arrhenius relationship, which states that the rate of vaccine degradation reactions are exponentially proportional to temperature [71]. In this way, we extrapolated the accelerated stability data collected at 40 °C and 60 °C to commonly used storage temperatures outlined by the International Council on Harmonization (ICH) [52] and for determination of vaccine vial monitor (VVM) designations [72]. ICH guidelines suggest several climactic zones with temperatures ranging from 5 °C to 30 °C, which are meant to mimic actual storage conditions around the world, while VVM designations are focused on duration of stability at 37˚C as a predictor of stability at lower temperatures. Our data (from Fig. 1) are shown alongside stability predictions at the recommended temperatures in Table 1.

**Table 1:**
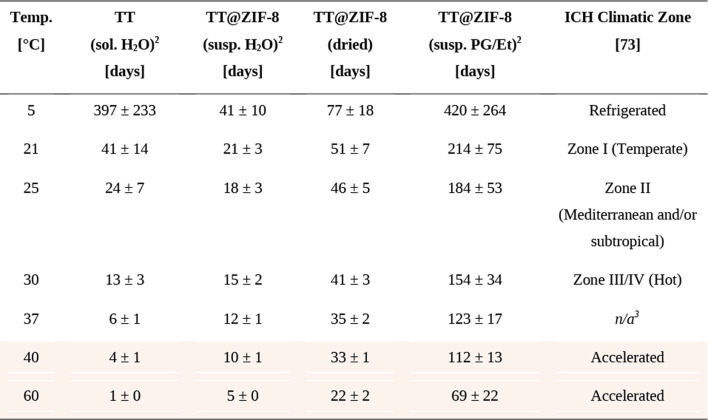
Predicted stability TT@ZIF-8 during storage at various temperatures as determined by arrhenius analysis.^1^

^1^Stability is reported as length of time of retention of at least 80% vaccine concentration (mean ± standard deviation). Shaded rows represent experimental data; unshaded rows are predicted by Arrhenius analysis

^2^sol. = solution. Susp. = suspension

^3^Although not a temperature corresponding to an ICH Climatic Zone, VVM designations are based in part on stability at 37˚C

The ICH recommends that vaccine formulations do not undergo a “significant change” in stability for 6–12 months in each climatic zone [73]. Because this definition varies across vaccines, we set a threshold of 80% stability retained (i.e., a formulation was considered to be unstable if it experienced a > 20% activity loss during storage. This threshold is a representative, likely conservative value, as WHO guidance indicates that a TT potency 50% lower than the minimum value is acceptable [74]. ICH also specifies storage humidity, but our vaccines were stored with desiccant maintaining very low humidity, which is how we expect commercial vaccines would be stored too. These predictions suggest that neither unencapsulated TT in water nor the aqueous TT@ZIF-8 formulation was sufficiently stable for one-year storage at any temperature, including refrigeration (Table 1). The dried formulation of TT@ZIF-8 was predicted to remain stable under refrigeration for approximately 2.5 months, but only retained sufficient activity at the various temperatures without refrigeration for less than 1.5 months.

In contrast, the non-aqueous suspension of TT@ZIF-8 in PG/Et was much more stable, exhibiting over one-year stability under refrigeration and more than half a year of stability at 21˚C (ICH climatic zone I) and 25˚C (ICH climatic zone II) (Table 1). Although falling below the half-year stability benchmark at higher temperatures, TT@ZIF-8 in PG/Et was predicted to be stable for approximately 5 and 4 months at 30˚C and 37˚C (corresponding to ICH climatic III/IV and VVM high stability), respectively. These data indicate that exclusion of water, which can degrade ZIF-8 and possibly TT as well, is important for TT@ZIF-8 stability. Improved drying to more fully exclude residual water from dried TT@ZIF-8 could enable further improvements.

Stability analysis at 37˚C is useful for VVM labeling [72], which can enable vaccine storage and distribution in a “controlled temperature chain,” allowing vaccines to be kept at non-refrigerated temperatures for a period of time. This could be during an (unintended) temperature excursion that would otherwise call for the vaccine to be discarded, or during vaccination campaigns necessitating transport to remote locations or other special settings that lack refrigeration. A designation of VVM 30 is the most stable category, corresponding to stability for 30 days at 37˚C (and 193 days at 25˚C, and 4 years at 5˚C) [72]. TT@ZIF-8 suspended in in PG/Et was predicted to meet the VVM 30 criteria at 37˚C and 25˚C, providing further evidence of the value of our MOF-based approach to vaccine stability. Dried TT@ZIF-8 met the VVM 30 criteria at 37˚C but not for 25˚C. TT@ZIF-8 suspended in water was predicted to be the least stable, and did not meet any of the VVM stability guidelines.

## Discussion

In this study, TT vaccine was encapsulated within ZIF-8 frameworks and characterized with microscopic, crystallographic, and spectroscopic techniques to confirm the formation of the TT@ZIF-8 biocomposite. The vaccine-ZIF composite was then prepared as liquid and solid formulations, and exposed to a variety of storage conditions, including elevated temperatures and freeze/thaw cycles. Through these experiments, we assessed the stability of a widely used, commercial vaccine antigen (i.e., TT obtained from SII) at elevated temperatures for accelerated testing up to 6 months. This enabled prediction of vaccine stability to meet requirements for storage in a controlled temperature chain or completely outside the cold chain.

Characterization of TT@ZIF-8 through pXRD, FTIR, SEM, and confocal microscopy provided strong evidence that TT was encapsulated throughout the ZIF structure, rather than just being adhered to the ZIF surface. Comparing the diffractograms for ZIF-8 and TT@ZIF-8 (Fig. 2a**)** suggested that incorporating TT into the MOF did not significantly alter the pure ZIF structure or crystallinity. FTIR spectrograms showed that the TT@ZIF-8 biocomposite contained both the characteristic proteinaceous peaks (Amide I and III) seen in TT and the characteristic ZIF plateau and peak owed to the methyl group and aromatic amine, respectively, composing the 2-methyl imidazole ligand (Fig. 2b).

Fluorescently-tagged TT was encapsulated within ZIF particles and examined with confocal microscopy, allowing visual confirmation of TT’s distribution throughout the biocomposite (Fig. 3g). Biocomposite particle size (50–700 μm) and shape were observed by SEM, where the rhombic dodecahedron shape characteristic of some ZIF-8 biocomposites was notably not observed, possibly due to the growth of many smaller crystals on surfaces of the largest particles (Fig. 3a-d). The methods we employed to confirm TT encapsulation were consistent with previous studies characterizing other encapsulated biomacromolecules [20, 30, 55, 75].

After confirming a TT release efficiency of ~ 60% from the ZIF by ELISA, biocomposites were subjected to accelerated stability studies at 40 °C and 60 °C, enabling stability prediction at lower temperatures through the Arrhenius relationship. This approach to predicting stability is commonly employed in the pharmaceutical industry [73], which is why we adopted it here. While these predictions have value, they still need to be confirmed by additional studies at the actual storage conditions of interest. Furthermore, this work did not study the release profile of TT from the MOF composite; as such, additional experiments should study this release in physiological conditions to better understand the behavior of injected TT@MOF *in vivo.*

The best stability results were obtained by suspending TT@ZIF-8 in a PG/Et mixture, which afforded remarkable stability retention (71% at 40 °C and 57% at 60 °C) after 6 months (Fig. 1a, c). Interestingly, the dried TT@ZIF-8 samples were much less stable (24% at 60 °C for 6 months, Fig. 1d) than the non-aqueous liquid formulation, suggesting that even the presence of a small amount of residual water moisture that was presumably present in the dried samples considerably affected biocomposite stability. At an extreme temperature of 120 °C, TT@ZIF-8 lost essentially all activity within 2 months (Fig. 5a). TT@ZIF-8 experienced no significant stability loss when exposed to five freeze/thaw cycles (Fig. 5b).

This study used unadjuvanted TT vaccine, whereas commercial formulations of TT-containing vaccines in clinical use generally contain an adjuvant, such as alum [9]. Previous studies using adjuvanted TT vaccine have reported greater thermostability than we found for unformulated TT in our study, due to the stabilizing effect of TT in the presence of certain adjuvants [76]. For example, Galazka et al. reported that the TT component of the adjuvanted DTP vaccine had no significant activity loss for 2–6 months at 37 °C and 2–4 weeks at 40 °C [9, 63], which is more stable than TT vaccine without adjuvant, but less stable than TT@ZIF-8 in our study.

Recent work has shown that ZIF-8 may provide an adjuvant effect for encapsulated antigens such as ovalbumin due to the metalloimmunological effects of zinc [77], providing further opportunities to improve both the stability and immunogenicity of ZIF-based biocomposites. Future studies may examine this adjuvant effect as well as potential effects on immune response caused by Zn-EDTA residues generated during MOF dissolution. These studies are important for translational applications, as the necessity to remove residues and/or purify MOF-encapsulated vaccines after disintegration might necessitate an additional preparatory step before vaccine administration.

Although TT encapsulated within ZIF-8 exhibited exceptional thermostability, the safety of such MOF formulations must be carefully considered. While some studies in literature have shown ZIF-8 to be non-toxic and non-accumulative at low doses [30], others have demonstrated cytotoxicity of ZIF-8 nanoparticles at higher doses (owed to zinc accumulation resulting in necrocytosis) [78]. Thus, further safety studies on ZIF-8 toxicity at the doses needed to stabilize TT and possibly other vaccines are required before broad application of this approach.

When considering future regulatory approaches and challenges (e.g., for product labels), we expect that a MOF-formulated TT vaccine product would report the amount of active antigen available after release from the ZIF structure. In the current formulation, this would amount to 60% of the TT added to the initial biomimetic mineralization reaction, representing the active antigen released from the MOF. This work found the variance in the 60% release efficiency to be 5–10%, suggesting that a post-release TT dose of 7.5 units would fall within the recommended range of 5–10 units in a TT vaccine [79]. From a regulatory standpoint, further studies would need to be conducted on the 40% “inactive” TT within the MOF biocomposite to confirm whether it would cause any immunogenic or other effect.

Extrapolation of these accelerated stability studies *via* the Arrhenius relationship suggested that TT@ZIF-8 suspended in the non-aqueous PG/Et solvent could meet ICH stability guidelines at 21 °C (climatic zone I) and 25˚C (ICH climatic zone II), and may satisfy VVM 30 requirements, highlighting the translational viability of these biocomposites for long-term vaccine storage with reduced or no need for refrigeration. While accelerated study at elevated temperature enables a mathematical prediction of stability at lower temperatures, further experiments are needed for confirmation. Reaction pathways for MOF dissolution and damage to TT antigenicity may vary with temperature, so instability mechanisms at higher temperatures may differ from those at lower ones, potentially over- or underestimating TT stability, necessitating additional research to confirm these results. Further formulation optimization could enable still greater stability, allowing non-refrigerated storage in additional climatic zones.

These findings are significant because vaccines, as well as biologic drugs in general, are usually stored at refrigerated temperature and sometimes require freezing due to the instability of proteins and other biomolecules and biomolecular structures [80, 81]. The associated cold-chain requirements result in extensive vaccine wastage due to cold-chain failures, increased cost of cold-chain infrastructure, and reduced vaccine access and vaccination coverage caused by lack of cold chain in low-resource settings [7]. Reduced reliance on the cold chain for vaccine storage and transport would have many benefits to public health, especially in developing countries.

TT vaccine is indicated for essentially every child as a six-dose regimen, followed by booster doses every 10 years and in the event of pregnancy or wound management [82]. However, despite availability of an effective vaccine, there are an estimated 1 million cases of tetanus resulting in 200,000 deaths per year [83]. This high caseload can be explained in part by only 84% coverage of a third dose of DTP vaccine worldwide in 2022 [3]; higher TT vaccination rates should reduce tetanus cases and deaths. Increased access to TT vaccine could be facilitated by storage and distribution of vaccine with reduced or no cold chain requirements, thereby enabling vaccine to reach the most under-served populations.

## Conclusion

Here, we reported that TT vaccine was encapsulated within ZIF-8 MOF, and that this encapsulation imparted significant thermostability to the embedded vaccine. TT was embedded within the ZIF-8 particle with an encapsulation efficiency of ~80% and did not interfere with the host material’s crystal structure. Dissolution of the TT@ZIF biocomposites with EDTA showed a release efficiency of ~60% from the framework.

In accelerated stability studies, TT@ZIF suspended in a PG/Et mixture was the most stable among various formulations, retaining 71% and 57% activity after 6 months of storage at 40 °C and 60 °C, respectively. In contrast, unencapsulated TT vaccine in water lost all potency after 3 months at 40 °C and 1 month at 60 °C, and unencapsulated TT in PG/Et lost potency even faster. Dried TT@ZIF powder was the second most stable formulation, retaining 31% and 24% activity after 6 months of storage at 40 °C and 60 °C, respectively, representing a marked stability improvement over lyophilized unencapsulated TT, which lost all activity within a week at either temperature. TT@ZIF suspended in water exhibited only a modest increase in stability compared to unencapsulated TT in water, presumably due to the relatively rapid degradation of the ZIF-8 particle by hydrolysis.

Arrhenius analysis of the accelerated stability data predicted that TT@ZIF suspended in PG/Et would retain at least 80% stability for more than 6 months at 21˚C and 25˚C, suggesting stability outside the cold chain in ICH climatic zones I and II, respectively, and remain stable for 4 months at 37 °C, indicating qualification for “controlled temperature chain” designation according to VVM criteria. Further formulation optimization (e.g., assessing other non-aqueous solvents instead of PG/Et to improve comfort during injection) and possible relaxation of the 80% stability requirement (which was selected as a representative example) could extend stability designations further.

Overall, we conclude that encapsulation of TT in ZIF-8 particles and storage in a non-aqueous environment can enable significant vaccine stability improvements for partial or complete vaccine removal from the cold chain, thereby increasing access and reducing cost of vaccination. Additional formulation improvements could further improve vaccine stability, immunogenicity, and clinical applicability.

## Data Availability

The data underlying this study are available in the published article.
